# Methyl 2-acetamido-2-(1-acetyl-3-hydr­oxy-2-oxoindolin-3-yl)propanoate

**DOI:** 10.1107/S1600536810007270

**Published:** 2010-03-03

**Authors:** Hoong-Kun Fun, Jia Hao Goh, Yang Liu, Yan Zhang

**Affiliations:** a X-ray Crystallography Unit, School of Physics, Universiti Sains Malaysia, 11800 USM, Penang, Malaysia; b School of Chemistry and Chemical Engineering, Nanjing University, Nanjing 210093, People’s Republic of China.

## Abstract

In the title isatin compound, C_16_H_18_N_2_O_6_, the pyrrolidine ring adopts an envelope conformation and is inclined at a dihedral angle of 7.31 (5)° with respect to the benzene ring. The acetyl group is disordered over two positions with refined occupancies of 0.503 (4) and 0.497 (4). These groups make dihedral angles of 12.6 (6) and 19.6 (7)° with the pyrrolidine ring. In the crystal structure, inter­molecular C—H⋯O hydrogen bonds link neighbouring mol­ecules into infinite chains along the *b* axis. These chains are further inter­connected by inter­molecular O—H⋯O hydrogen bonds into two-dimensional arrays parallel to the *bc* plane. Weak inter­molecular C—H⋯π inter­actions further stabilize the crystal structure.

## Related literature

For general background to and applications of isatin derivatives, see: Chu *et al.* (2007 ▶); Glover & Bhattacharya (1991 ▶); Gursoy & Karali (1996 ▶); Pandeya *et al.* (1998 ▶); Patel *et al.* (2006 ▶); Popp (1975 ▶); Shvekhgeimer (1996 ▶); Sriram *et al.* (2006 ▶); Verma *et al.* (2004 ▶); Vine *et al.* (2007 ▶). For photoreactions of *N*-acetyl­isatin, see: Zhang *et al.* (2004 ▶). For ring conformations, see: Cremer & Pople (1975 ▶). For related structures, see: Usman *et al.* (2001 ▶, 2002**a* ▶,b* ▶). For the stability of the temperature controller used for the data collection, see: Cosier & Glazer (1986 ▶).
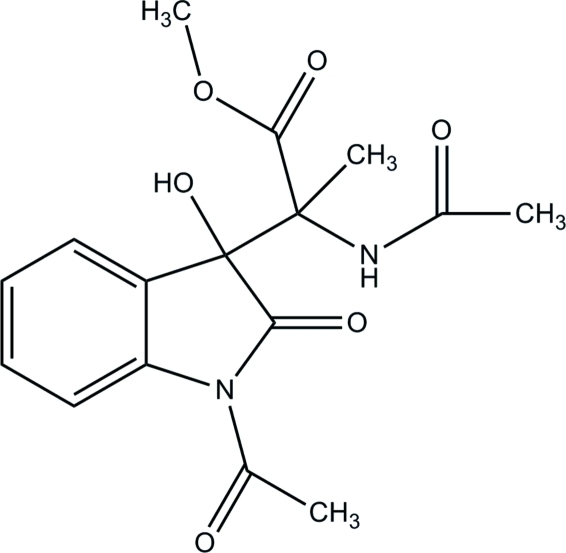


## Experimental

### 

#### Crystal data

C_16_H_18_N_2_O_6_
*M* *_r_* = 334.32
Monoclinic, 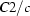

*a* = 28.4345 (6) Å
*b* = 8.3396 (2) Å
*c* = 14.3779 (3) Å
β = 114.351 (2)°
*V* = 3106.15 (12) Å^3^
*Z* = 8
Mo *K*α radiation
μ = 0.11 mm^−1^
*T* = 100 K
0.47 × 0.37 × 0.26 mm

#### Data collection

Bruker SMART APEX Duo CCD area-detector diffractometer
Absorption correction: multi-scan (*SADABS*; Bruker, 2009 ▶) *T*_min_ = 0.950, *T*_max_ = 0.972
39997 measured reflections
5705 independent reflections
4854 reflections with *I* > 2σ(*I*)
*R*_int_ = 0.033

#### Refinement

*R*[*F* ^2^ > 2σ(*F* ^2^)] = 0.045
*wR*(*F* ^2^) = 0.126
*S* = 1.03
5705 reflections
258 parameters
H atoms treated by a mixture of independent and constrained refinement
Δρ_max_ = 0.48 e Å^−3^
Δρ_min_ = −0.36 e Å^−3^

### 

Data collection: *APEX2* (Bruker, 2009 ▶); cell refinement: *SAINT* (Bruker, 2009 ▶); data reduction: *SAINT*; program(s) used to solve structure: *SHELXTL* (Sheldrick, 2008 ▶); program(s) used to refine structure: *SHELXTL*; molecular graphics: *SHELXTL*; software used to prepare material for publication: *SHELXTL* and *PLATON* (Spek, 2009 ▶).

## Supplementary Material

Crystal structure: contains datablocks global, I. DOI: 10.1107/S1600536810007270/sj2735sup1.cif

Structure factors: contains datablocks I. DOI: 10.1107/S1600536810007270/sj2735Isup2.hkl

Additional supplementary materials:  crystallographic information; 3D view; checkCIF report

## Figures and Tables

**Table 1:** Hydrogen-bond geometry (Å, °) *Cg*1 is the centroid of the C1–C6 benzene ring.

*D*—H⋯*A*	*D*—H	H⋯*A*	*D*⋯*A*	*D*—H⋯*A*
O1—H1*O*1⋯O6^i^	0.875 (17)	1.769 (17)	2.6391 (10)	172 (2)
C3—H3*A*⋯O1^ii^	0.93	2.58	3.4098 (12)	150
C15—H15*C*⋯*Cg*1^iii^	0.96	2.96	3.9104 (11)	169

Symmetry codes: (i) 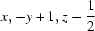; (ii) 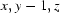; (iii) 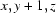.

**Fig. 1.**
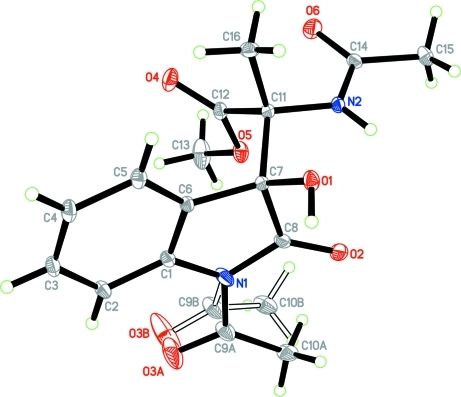
The structure of the title compound, showing the atom numbering scheme. Displacement ellipsoids are drawn at the 30% probability level. Bonds to atoms of the minor disordered component are drawn as open lines.

**Fig. 2.**
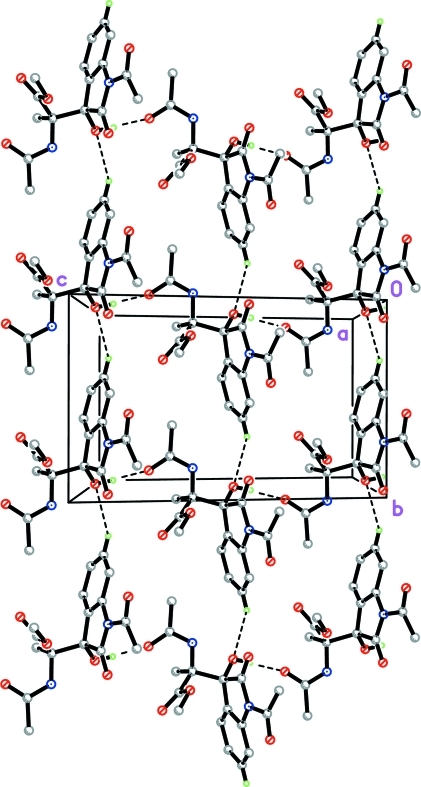
The crystal structure of the title compound, viewed along the a axis, showing a two-dimensional array parallel to the bc plane. Only the major disordered components are shown. H atoms not involved in intermolecular interactions (dashed lines) have been omitted for clarity.

## supplementary crystallographic information

### Comment

Isatin (1*H*-indole-2,3-dione) was first discovered by Erdmann and Laurent in 1841 (Popp, 1975). Isatin and its derivatives are versatile molecules and possess a wide range of activities, especially in the biological and pharmaceutical fields. They are also basic structural units and important synthetic precursors of many naturally occuring alkaloids (Shvekhgeimer, 1996). Several of its derivatives were reported to exhibit a wide range of promising pharmacodynamic profiles displaying anti-convulsant (Gursoy & Karali, 1996; Verma *et al.*, 2004), anti-HIV (Pandeya *et al.*, 1998), cytotoxic (Vine *et al.*, 2007), tuberculostatic (Sriram *et al.*, 2006) and anti-microbial (Patel *et al.*, 2006) activities. At millimolar concentrations, isatin has been found to inhibit different enzymes, an effect that may contribute to its anti-infective actions (Glover & Bhattacharya, 1991).  Recently, a number of isatin-based compounds are reported as inhibitors of caspase-3 and caspase-7 (Chu *et al.*, 2007). Photoreactions of N-acetylisatin with various species have also been of research interest (Zhang *et al.* 2004). Due to the importance of the isatin derivatives, the crystal structure of the biologically active title compound is reported in this paper.

In the title isatin compound (Fig. 1), atoms C7 and C11 are chiral centers. The indoline moiety is not planar, which is inconsistent with those related structures previously studied (Usman *et al.*, 2001, 2002*a,b*). The pyrrolidine ring (N1/C1/C6-C8) of the indoline moiety adopts an envelope conformation, with puckering parameters of Q = 0.1013 (10) Å and φ = 132.3 (6)° (Cremer & Pople, 1975) and is inclined at a dihedral angle of 7.31 (5) with the (C1-C6) benzene ring. The acetyl group is disordered over two positions with a refined occupancy ratio of 0.503 (4):0.497 (4). The major (O3A/C9A/C10A) and minor (O3B/C9B/C10B) disorder components make dihedral angles of 12.6 (6) and 19.6 (7)°, respectively, to the attached pyrrolidine ring. The bond lengths are within normal ranges and agree well with those in related indoline structures (Usman *et al.*, 2001, 2002*a,b*).

In the crystal structure (Fig. 2), intermolecular C3—H3A···O1 hydrogen bonds (Table 1) link neighbouring molecules into infinite chains along the *b* axis. These chains are further interconnected by intermolecular O1—H1O1···O6 hydrogen bonds (Table 1) into two-dimensional arrays parallel to the *bc* plane. Weak intermolecular C15—H15C···*Cg*1 interactions (Table 1) involving the centroid of C1-C6 benzene ring further stabilize the crystal structure.

### Experimental

The title compound was obtained in the reaction between N-acetylisatin and 2,4-dimethyl-5-methyloxy-oxazole. The compound was purified by flash column chromatography. X-ray quality single crystals of the title compound were obtained from slow evaporation of a solution of chloroform and petroleum ether (1:3; v:v). *M.p.* 431–434 K.

### Refinement

Atoms H1O1 and H1N1 were located from difference Fourier map and allowed to refine freely. All other hydrogen atoms were placed in their calculated positions, with C—H = 0.93 or 0.96 Å, and refined using a riding model, with *U*_iso_(H) = 1.2 or 1.5*U*_eq_(C). The acetyl group is disordered over two positions with a refined occupancy ratio of 0.503 (4):0.497 (4). Three short intermolecular interactions involving the major disordered components [C9A···C9A = 3.027 (6) Å, C9A···C10A = 2.913 (4) Å, C10A···C10A = 2.621 (3) Å] which are shorter than the sum of the van der Waals radius of the carbon atom are observed.

### Crystal data

**Table:**

C_16_H_18_N_2_O_6_	*F*(000) = 1408
*M**_r_* = 334.32	*D*_x_ = 1.430 Mg m^−^^3^
Monoclinic, *C*2/*c*	Mo *K*α radiation, λ = 0.71073 Å
Hall symbol: -C 2yc	Cell parameters from 9185 reflections
*a* = 28.4345 (6) Å	θ = 2.6–32.6°
*b* = 8.3396 (2) Å	µ = 0.11 mm^−^^1^
*c* = 14.3779 (3) Å	*T* = 100 K
β = 114.351 (2)°	Block, colourless
*V* = 3106.15 (12) Å^3^	0.47 × 0.37 × 0.26 mm
*Z* = 8	

### Data collection

**Table:**

Bruker SMART APEX Duo CCD area-detector diffractometer	5705 independent reflections
Radiation source: fine-focus sealed tube	4854 reflections with *I* > 2σ(*I*)
graphite	*R*_int_ = 0.033
φ and ω scans	θ_max_ = 32.7°, θ_min_ = 1.6°
Absorption correction: multi-scan (*SADABS*; Bruker, 2009)	*h* = −42→43
*T*_min_ = 0.950, *T*_max_ = 0.972	*k* = −12→12
39997 measured reflections	*l* = −21→21

### Refinement

**Table:**

Refinement on *F*^2^	Primary atom site location: structure-invariant direct methods
Least-squares matrix: full	Secondary atom site location: difference Fourier map
*R*[*F*^2^ > 2σ(*F*^2^)] = 0.045	Hydrogen site location: inferred from neighbouring sites
*wR*(*F*^2^) = 0.126	H atoms treated by a mixture of independent and constrained refinement
*S* = 1.03	*w* = 1/[σ^2^(*F*_o_^2^) + (0.0685*P*)^2^ + 1.8239*P*] where *P* = (*F*_o_^2^ + 2*F*_c_^2^)/3
5705 reflections	(Δ/σ)_max_ < 0.001
258 parameters	Δρ_max_ = 0.48 e Å^−^^3^
0 restraints	Δρ_min_ = −0.36 e Å^−^^3^

### Special details

**Table:**

Experimental. The crystal was placed in the cold stream of an Oxford Cryosystems Cobra open-flow nitrogen cryostat (Cosier & Glazer, 1986) operating at 100.0 (1)K.
Geometry. All esds (except the esd in the dihedral angle between two l.s. planes) are estimated using the full covariance matrix. The cell esds are taken into account individually in the estimation of esds in distances, angles and torsion angles; correlations between esds in cell parameters are only used when they are defined by crystal symmetry. An approximate (isotropic) treatment of cell esds is used for estimating esds involving l.s. planes.
Refinement. Refinement of F^2^ against ALL reflections. The weighted R-factor wR and goodness of fit S are based on F^2^, conventional R-factors R are based on F, with F set to zero for negative F^2^. The threshold expression of F^2^ > 2sigma(F^2^) is used only for calculating R-factors(gt) etc. and is not relevant to the choice of reflections for refinement. R-factors based on F^2^ are statistically about twice as large as those based on F, and R- factors based on ALL data will be even larger.

### Fractional atomic coordinates and isotropic or equivalent isotropic displacement parameters (Å^2^)

**Table:**

	*x*	*y*	*z*	*U*_iso_*/*U*_eq_	Occ. (<1)
O1	0.18064 (3)	0.48686 (8)	−0.01504 (5)	0.02081 (14)	
O2	0.07223 (3)	0.53296 (9)	−0.06871 (6)	0.02680 (16)	
O4	0.16093 (3)	0.21913 (9)	0.24294 (6)	0.02902 (17)	
O5	0.08943 (3)	0.35325 (9)	0.14131 (6)	0.02432 (15)	
O6	0.15655 (4)	0.58921 (10)	0.29251 (6)	0.03130 (18)	
N1	0.07133 (3)	0.25274 (11)	−0.07079 (8)	0.0300 (2)	
N2	0.15337 (3)	0.59960 (9)	0.13473 (6)	0.01608 (14)	
C1	0.11099 (4)	0.13422 (11)	−0.04580 (7)	0.01937 (17)	
C2	0.10601 (4)	−0.02706 (11)	−0.07223 (7)	0.02217 (18)	
H2A	0.0737	−0.0746	−0.1052	0.027*	
C3	0.15133 (4)	−0.11480 (11)	−0.04742 (7)	0.02182 (18)	
H3A	0.1492	−0.2230	−0.0643	0.026*	
C4	0.19967 (4)	−0.04441 (11)	0.00189 (8)	0.02254 (18)	
H4A	0.2293	−0.1054	0.0169	0.027*	
C5	0.20390 (4)	0.11744 (11)	0.02897 (7)	0.01982 (17)	
H5A	0.2361	0.1650	0.0621	0.024*	
C6	0.15917 (3)	0.20604 (10)	0.00553 (6)	0.01592 (15)	
C7	0.15271 (3)	0.38261 (10)	0.02069 (6)	0.01522 (15)	
C8	0.09386 (4)	0.40548 (11)	−0.04325 (7)	0.02089 (17)	
O3A	0.0031 (3)	0.0870 (9)	−0.1455 (7)	0.068 (2)	0.503 (4)
C9A	0.01989 (9)	0.2274 (3)	−0.1348 (3)	0.0290 (5)	0.503 (4)
C10A	−0.01576 (8)	0.3669 (3)	−0.17624 (17)	0.0287 (5)	0.503 (4)
H10A	−0.0488	0.3293	−0.2242	0.043*	0.503 (4)
H10B	−0.0016	0.4393	−0.2099	0.043*	0.503 (4)
H10C	−0.0198	0.4216	−0.1212	0.043*	0.503 (4)
O3B	0.0045 (3)	0.0780 (10)	−0.1114 (8)	0.097 (3)	0.497 (4)
C9B	0.01733 (9)	0.2087 (3)	−0.0877 (3)	0.0343 (6)	0.497 (4)
C10B	−0.01742 (8)	0.3377 (3)	−0.0815 (2)	0.0339 (6)	0.497 (4)
H10D	−0.0347	0.3870	−0.1473	0.051*	0.497 (4)
H10E	0.0026	0.4169	−0.0328	0.051*	0.497 (4)
H10F	−0.0425	0.2924	−0.0605	0.051*	0.497 (4)
C11	0.16854 (3)	0.43179 (10)	0.13503 (6)	0.01508 (14)	
C12	0.14032 (4)	0.32345 (11)	0.18209 (7)	0.01931 (16)	
C13	0.05969 (6)	0.25468 (15)	0.18069 (14)	0.0438 (3)	
H13A	0.0236	0.2769	0.1434	0.066*	
H13B	0.0696	0.2785	0.2516	0.066*	
H13C	0.0661	0.1435	0.1731	0.066*	
C14	0.15038 (3)	0.66917 (11)	0.21616 (7)	0.01811 (16)	
C15	0.13850 (4)	0.84545 (11)	0.20910 (8)	0.02441 (19)	
H15A	0.1577	0.8950	0.2741	0.037*	
H15B	0.1022	0.8606	0.1901	0.037*	
H15C	0.1480	0.8934	0.1586	0.037*	
C16	0.22685 (4)	0.41480 (12)	0.19614 (7)	0.02257 (18)	
H16A	0.2363	0.4557	0.2639	0.034*	
H16B	0.2446	0.4743	0.1632	0.034*	
H16C	0.2363	0.3037	0.1997	0.034*	
H1O1	0.1751 (7)	0.467 (2)	−0.0785 (13)	0.043 (4)*	
H1N2	0.1491 (6)	0.658 (2)	0.0803 (13)	0.037 (4)*	

### Atomic displacement parameters (Å^2^)

**Table:**

	*U*^11^	*U*^22^	*U*^33^	*U*^12^	*U*^13^	*U*^23^
O1	0.0311 (3)	0.0154 (3)	0.0206 (3)	−0.0004 (2)	0.0154 (3)	0.0001 (2)
O2	0.0279 (4)	0.0212 (3)	0.0225 (3)	0.0096 (3)	0.0016 (3)	−0.0024 (3)
O4	0.0417 (4)	0.0207 (3)	0.0283 (4)	0.0071 (3)	0.0181 (3)	0.0091 (3)
O5	0.0234 (3)	0.0183 (3)	0.0349 (4)	−0.0014 (2)	0.0156 (3)	0.0029 (3)
O6	0.0504 (5)	0.0266 (4)	0.0169 (3)	0.0064 (3)	0.0138 (3)	0.0000 (3)
N1	0.0182 (4)	0.0203 (4)	0.0376 (5)	0.0029 (3)	−0.0025 (3)	−0.0127 (3)
N2	0.0207 (3)	0.0119 (3)	0.0159 (3)	0.0007 (2)	0.0078 (3)	−0.0004 (2)
C1	0.0213 (4)	0.0154 (4)	0.0192 (4)	0.0019 (3)	0.0061 (3)	−0.0038 (3)
C2	0.0280 (4)	0.0162 (4)	0.0220 (4)	−0.0012 (3)	0.0099 (3)	−0.0047 (3)
C3	0.0344 (5)	0.0127 (3)	0.0223 (4)	0.0018 (3)	0.0157 (4)	−0.0003 (3)
C4	0.0289 (4)	0.0154 (4)	0.0277 (4)	0.0062 (3)	0.0162 (4)	0.0022 (3)
C5	0.0221 (4)	0.0163 (4)	0.0232 (4)	0.0034 (3)	0.0114 (3)	0.0008 (3)
C6	0.0194 (4)	0.0129 (3)	0.0154 (3)	0.0019 (3)	0.0072 (3)	−0.0005 (3)
C7	0.0176 (3)	0.0124 (3)	0.0147 (3)	0.0015 (3)	0.0058 (3)	−0.0008 (3)
C8	0.0203 (4)	0.0183 (4)	0.0178 (4)	0.0034 (3)	0.0015 (3)	−0.0054 (3)
O3A	0.031 (2)	0.0249 (15)	0.107 (3)	−0.0043 (12)	−0.012 (2)	−0.0154 (19)
C9A	0.0171 (9)	0.0259 (10)	0.0374 (14)	−0.0030 (7)	0.0044 (9)	−0.0101 (10)
C10A	0.0171 (8)	0.0334 (11)	0.0279 (10)	0.0015 (7)	0.0014 (7)	−0.0044 (8)
O3B	0.024 (2)	0.050 (3)	0.204 (9)	−0.0125 (18)	0.035 (4)	−0.065 (4)
C9B	0.0188 (9)	0.0314 (12)	0.0486 (17)	−0.0025 (8)	0.0096 (11)	−0.0163 (12)
C10B	0.0182 (9)	0.0401 (13)	0.0380 (12)	0.0013 (8)	0.0063 (8)	−0.0151 (10)
C11	0.0167 (3)	0.0129 (3)	0.0143 (3)	0.0010 (3)	0.0051 (3)	−0.0006 (3)
C12	0.0261 (4)	0.0140 (3)	0.0201 (4)	0.0012 (3)	0.0119 (3)	0.0001 (3)
C13	0.0427 (7)	0.0260 (5)	0.0786 (10)	−0.0036 (5)	0.0409 (7)	0.0095 (6)
C14	0.0189 (4)	0.0166 (4)	0.0173 (4)	−0.0010 (3)	0.0060 (3)	−0.0042 (3)
C15	0.0282 (4)	0.0160 (4)	0.0318 (5)	−0.0007 (3)	0.0152 (4)	−0.0060 (3)
C16	0.0175 (4)	0.0238 (4)	0.0207 (4)	0.0022 (3)	0.0021 (3)	−0.0036 (3)

### Geometric parameters (Å, °)

**Table:**

O1—C7	1.4095 (11)	C7—C8	1.5533 (12)
O1—H1O1	0.875 (18)	C7—C11	1.5695 (12)
O2—C8	1.2070 (11)	O3A—C9A	1.250 (8)
O4—C12	1.2013 (11)	C9A—C10A	1.496 (3)
O5—C12	1.3416 (12)	C10A—H10A	0.9600
O5—C13	1.4514 (13)	C10A—H10B	0.9600
O6—C14	1.2336 (12)	C10A—H10C	0.9600
N1—C9A	1.386 (2)	O3B—C9B	1.156 (9)
N1—C8	1.4074 (13)	C9B—C10B	1.488 (3)
N1—C1	1.4295 (12)	C10B—H10D	0.9600
N1—C9B	1.498 (3)	C10B—H10E	0.9600
N2—C14	1.3404 (11)	C10B—H10F	0.9600
N2—C11	1.4639 (11)	C11—C16	1.5298 (12)
N2—H1N2	0.888 (17)	C11—C12	1.5379 (12)
C1—C2	1.3890 (12)	C13—H13A	0.9600
C1—C6	1.3952 (12)	C13—H13B	0.9600
C2—C3	1.3944 (14)	C13—H13C	0.9600
C2—H2A	0.9300	C14—C15	1.5025 (13)
C3—C4	1.3904 (14)	C15—H15A	0.9600
C3—H3A	0.9300	C15—H15B	0.9600
C4—C5	1.3962 (13)	C15—H15C	0.9600
C4—H4A	0.9300	C16—H16A	0.9600
C5—C6	1.3871 (12)	C16—H16B	0.9600
C5—H5A	0.9300	C16—H16C	0.9600
C6—C7	1.5109 (11)		
			
C7—O1—H1O1	111.9 (12)	N1—C9A—C10A	120.17 (19)
C12—O5—C13	114.94 (9)	O3B—C9B—C10B	124.2 (4)
C9A—N1—C8	123.92 (13)	O3B—C9B—N1	117.2 (4)
C9A—N1—C1	124.67 (12)	C10B—C9B—N1	118.4 (2)
C8—N1—C1	109.55 (8)	C9B—C10B—H10D	109.5
C9A—N1—C9B	28.89 (14)	C9B—C10B—H10E	109.5
C8—N1—C9B	125.78 (12)	H10D—C10B—H10E	109.5
C1—N1—C9B	121.41 (14)	C9B—C10B—H10F	109.5
C14—N2—C11	122.41 (7)	H10D—C10B—H10F	109.5
C14—N2—H1N2	119.8 (11)	H10E—C10B—H10F	109.5
C11—N2—H1N2	117.5 (11)	N2—C11—C16	109.79 (7)
C2—C1—C6	121.84 (8)	N2—C11—C12	110.88 (7)
C2—C1—N1	128.31 (9)	C16—C11—C12	109.35 (7)
C6—C1—N1	109.69 (8)	N2—C11—C7	106.85 (7)
C1—C2—C3	117.32 (9)	C16—C11—C7	110.62 (7)
C1—C2—H2A	121.3	C12—C11—C7	109.33 (7)
C3—C2—H2A	121.3	O4—C12—O5	124.52 (9)
C4—C3—C2	121.60 (9)	O4—C12—C11	124.03 (9)
C4—C3—H3A	119.2	O5—C12—C11	111.30 (7)
C2—C3—H3A	119.2	O5—C13—H13A	109.5
C3—C4—C5	120.25 (9)	O5—C13—H13B	109.5
C3—C4—H4A	119.9	H13A—C13—H13B	109.5
C5—C4—H4A	119.9	O5—C13—H13C	109.5
C6—C5—C4	118.84 (9)	H13A—C13—H13C	109.5
C6—C5—H5A	120.6	H13B—C13—H13C	109.5
C4—C5—H5A	120.6	O6—C14—N2	120.42 (8)
C5—C6—C1	120.13 (8)	O6—C14—C15	122.31 (9)
C5—C6—C7	129.64 (8)	N2—C14—C15	117.26 (8)
C1—C6—C7	110.08 (7)	C14—C15—H15A	109.5
O1—C7—C6	115.36 (7)	C14—C15—H15B	109.5
O1—C7—C8	109.87 (7)	H15A—C15—H15B	109.5
C6—C7—C8	101.52 (7)	C14—C15—H15C	109.5
O1—C7—C11	105.03 (7)	H15A—C15—H15C	109.5
C6—C7—C11	113.98 (7)	H15B—C15—H15C	109.5
C8—C7—C11	111.19 (7)	C11—C16—H16A	109.5
O2—C8—N1	126.58 (9)	C11—C16—H16B	109.5
O2—C8—C7	125.27 (9)	H16A—C16—H16B	109.5
N1—C8—C7	108.05 (7)	C11—C16—H16C	109.5
O3A—C9A—N1	118.0 (4)	H16A—C16—H16C	109.5
O3A—C9A—C10A	121.2 (4)	H16B—C16—H16C	109.5
			
C9A—N1—C1—C2	−5.6 (3)	C8—N1—C9A—O3A	−173.0 (6)
C8—N1—C1—C2	−170.47 (10)	C1—N1—C9A—O3A	24.2 (7)
C9B—N1—C1—C2	28.8 (2)	C9B—N1—C9A—O3A	−69.1 (6)
C9A—N1—C1—C6	169.8 (2)	C8—N1—C9A—C10A	−2.2 (4)
C8—N1—C1—C6	4.91 (12)	C1—N1—C9A—C10A	−165.0 (2)
C9B—N1—C1—C6	−155.78 (19)	C9B—N1—C9A—C10A	101.7 (4)
C6—C1—C2—C3	−1.38 (14)	C9A—N1—C9B—O3B	82.3 (7)
N1—C1—C2—C3	173.51 (10)	C8—N1—C9B—O3B	179.1 (6)
C1—C2—C3—C4	0.06 (14)	C1—N1—C9B—O3B	−23.5 (7)
C2—C3—C4—C5	0.73 (15)	C9A—N1—C9B—C10B	−92.8 (5)
C3—C4—C5—C6	−0.20 (14)	C8—N1—C9B—C10B	4.0 (4)
C4—C5—C6—C1	−1.09 (13)	C1—N1—C9B—C10B	161.4 (2)
C4—C5—C6—C7	−176.31 (9)	C14—N2—C11—C16	−75.49 (10)
C2—C1—C6—C5	1.93 (14)	C14—N2—C11—C12	45.45 (11)
N1—C1—C6—C5	−173.81 (9)	C14—N2—C11—C7	164.50 (8)
C2—C1—C6—C7	178.01 (8)	O1—C7—C11—N2	60.42 (8)
N1—C1—C6—C7	2.27 (11)	C6—C7—C11—N2	−172.37 (7)
C5—C6—C7—O1	49.26 (12)	C8—C7—C11—N2	−58.36 (9)
C1—C6—C7—O1	−126.33 (8)	O1—C7—C11—C16	−59.04 (9)
C5—C6—C7—C8	167.97 (9)	C6—C7—C11—C16	68.17 (9)
C1—C6—C7—C8	−7.62 (9)	C8—C7—C11—C16	−177.83 (7)
C5—C6—C7—C11	−72.40 (12)	O1—C7—C11—C12	−179.52 (7)
C1—C6—C7—C11	112.01 (8)	C6—C7—C11—C12	−52.31 (9)
C9A—N1—C8—O2	1.6 (3)	C8—C7—C11—C12	61.69 (9)
C1—N1—C8—O2	166.66 (10)	C13—O5—C12—O4	3.57 (15)
C9B—N1—C8—O2	−33.7 (3)	C13—O5—C12—C11	179.33 (9)
C9A—N1—C8—C7	−174.8 (2)	N2—C11—C12—O4	−135.23 (9)
C1—N1—C8—C7	−9.79 (12)	C16—C11—C12—O4	−14.03 (12)
C9B—N1—C8—C7	149.9 (2)	C7—C11—C12—O4	107.22 (10)
O1—C7—C8—O2	−43.51 (12)	N2—C11—C12—O5	48.98 (10)
C6—C7—C8—O2	−166.08 (10)	C16—C11—C12—O5	170.18 (7)
C11—C7—C8—O2	72.33 (12)	C7—C11—C12—O5	−68.56 (9)
O1—C7—C8—N1	133.01 (9)	C11—N2—C14—O6	−5.74 (14)
C6—C7—C8—N1	10.43 (10)	C11—N2—C14—C15	175.23 (8)
C11—C7—C8—N1	−111.16 (9)		

### Hydrogen-bond geometry (Å, °)

**Table:**

Cg1 is the centroid of the C1–C6 benzene ring.

**Table:**

*D*—H···*A*	*D*—H	H···*A*	*D*···*A*	*D*—H···*A*
O1—H1O1···O6^i^	0.875 (17)	1.769 (17)	2.6391 (10)	172 (2)
C3—H3A···O1^ii^	0.93	2.58	3.4098 (12)	150
C15—H15C···Cg1^iii^	0.96	2.96	3.9104 (11)	169

Symmetry codes: (i) *x*, −*y*+1, *z*−1/2; (ii) *x*, *y*−1, *z*; (iii) *x*, *y*+1, *z*.
